# Pitfalls with Vitamin D Research in Musculoskeletal Disorders and Recommendations on How to Avoid Them

**DOI:** 10.4274/jcrpe.galenos.2019.2019.0007

**Published:** 2019-09-03

**Authors:** Gary M. Kiebzak, Kevin M. Neal, Pooya Hosseinzadeh, Robert C. Olney, Michael A. Levine

**Affiliations:** 1 Nemours Children’s Hospital and Specialty Care, Department of Orthopedics and Sports Medicine, Florida, USA; 2 Nemours Children’s Specialty Care, Department of Orthopedic Surgery, Florida, USA; 3 Washington University Faculty of Medicine, Department of Orthopaedic Surgery, 1 Children’s Place, Missouri, USA; 4 Nemours Children’s Specialty Care, Department of Endocrinology, Florida, USA; 5 Children’s Hospital of Philadelphia, Center for Bone Health, Division of Endocrinology and Diabetes, Pennsylvania, USA

**Keywords:** Vitamin D, 25-hydroxyvitamin D, deficiency

## Abstract

Reports suggesting that vitamin D may have extraskeletal roles have renewed interest in vitamin D research and stimulated publication of an increasing number of new studies each year. These studies typically assess vitamin D status by measuring the blood concentration of 25-hydroxyvitamin D [25(OH)D], the principal circulating metabolite of vitamin D. Unfortunately, variations in assay format, inconsistency in interpreting 25(OH)D concentrations, cohort bias (age, body mass index, race, season of measurements etc.) and failure to measure critical variables needed to interpret study results, makes interpreting results and comparing studies difficult. Further, variation in reporting results (reporting mean values vs. percent of the cohort that is deficient, no clear statement as to clinical relevance of effect size, etc.) further limits interstudy analyses. In this paper, we discuss many common pitfalls in vitamin D research. We also provide recommendations on avoiding these pitfalls and suggest guidelines to enhance consistency in reporting results.

## Introduction

Interest in nonconventional actions of vitamin D remains high many years after the first reports linking vitamin D to a variety of extraskeletal actions. According to a Pubmed.gov search on the term “vitamin D” (December 2018) there were 4497 publications in 2018, and that does not include books, symposium proceedings or media articles on vitamin D. This interest naturally extends to pediatric orthopedic conditions, where studies have attempted to identify associations between vitamin D status and fracture risk, fracture severity, fracture healing, and skeletal disorders such as adolescent idiopathic scoliosis (AIS). Studies that assess vitamin D status do so by measuring the concentration of 25-hydroxyvitamin D [25(OH)D] in blood. This is because 25(OH)D is the principal circulating form of vitamin D, and is the precursor to the biologically active metabolite, 1,25-dihydroxyvitamin D [1,25(OH)D] (1,2). The concentration of 25(OH)D in blood reflects both the amount of parent vitamin D_3_ that is generated in the skin upon exposure to ultraviolet B radiation as well as the amounts of vitamin D_2_ and D_3_ that are obtained from the diet and vitamin supplements. Despite enormous interest in vitamin D research, there are many pitfalls that cloud interpretation of study results. This is due in part to the many variables that affect 25(OH)D concentration.

Our objective was to identify and discuss common design and data presentation problems in vitamin D study results. Reviews of the pediatric vitamin D literature, namely studies associating vitamin D status to fracture risk and studies assessing vitamin D status in AIS, are used as examples of the lack of clarity in presentation of study results. Finally, we will make recommendations about how these common pitfalls can be avoided.

## Categories of Pitfalls

Common problems that confound vitamin D research studies can be divided into three categories: 1) design issues, 2) inconsistent presentation of results, and 3) failure to account for variables that are known to influence serum 25(OH)D concentrations. In addition, a lack of assay standardization further confuses the picture, particularly when reports do not provide adequate information about how 25(OH)D was assayed. When such problems are present, they can make it difficult to generate direct comparisons with other studies and can confound interpretation of results.

As an example, we reviewed all known papers reporting 25(OH)D in patients with AIS (n=5) (3,4,5,6,7). Only two papers indicated the race/ethnic distributions in the cohort. All five papers reported the mean ± standard deviation (SD) for 25(OH)D but only two reported percent deficiency. Between these two reports, each used a different definition of deficiency. None of the papers accounted for use of vitamin supplements nor the use of sunscreen. Only two studies had the same inclusion criteria for the Cobb angle (thus, the severity of scoliosis varied). One paper did not report the type of assay used. Of the other four papers, three different assay systems were used. Individually, all the studies are interesting and make legitimate contributions, however, they are not directly comparable nor could they be used in a meta-analysis, in part because of the variability in study design and data presentation.

## Design Problems

Research studies should be hypothesis driven and not simply reporting measurement results. Stating *a priori* whether vitamin D status is hypothesized as a causative factor in the condition being studied, is secondary to the condition itself (i.e., the condition impacts vitamin D status), or has no relation to the condition, would help frame interpretation of results. Also, it should be stated *a priori* what difference in the magnitude of percent deficiency or 25(OH)D concentration between two comparative groups is considered to be clinically meaningful. For example, “We hypothesize that low 25(OH)D is a causative factor in spinal curve progression in AIS manifest by at least a 30% difference in 25(OH)D concentration compared to controls”. Such a statement would temper overinterpretation of clinically insignificant differences (despite statistically significant differences).

The blood level of 25(OH)D can change substantially in a very short period of time (literally within minutes) after exposure to sunlight, or over a longer time period with the use of oral supplements (2,8,9). Therefore, studies must be designed so that measurement of 25(OH)D occurs at time points that are relevant to the development of the study endpoints. For example, if attempting to correlate fracture risk with vitamin D status, the 25(OH)D should be measured within a very short time period after the patient presents with the fracture. For studies in which an effect might take place over a protracted period of time such as bone mineral density or muscle mass changes, or the effect of a treatment or procedure that may last many months, it would be appropriate to verify *chronic* vitamin D status. This might require repetitive determination of 25(OH)D levels over an interval of time that corresponds to the duration of time necessary for the outcome to be realized. This is because 25(OH)D levels over time are susceptible to the phenomenon of regression to the mean, whereby a patient may be deficient on one measurement, but insufficient or normal at a subsequent measurement (due perhaps as a consequence of season, lifestyle factors, assay variability, etc.). For example, if assessing the effect of vitamin D status on height over three years, it would be inappropriate to measure a single 25(OH)D concentration at the beginning of the study and then assume that this is a valid reflection of vitamin D status over the entire three year period of study.

Failure to measure all critical variables may confound interpretation of results (Table 1; variables affecting 25(OH)D concentration will be discussed further below). For example, if a study hypothesis states that vitamin D status impacts spinal curve progression in scoliosis or is a factor in fracture risk, then it is important to not only measure known variables that affect 25(OH)D concentration [such as body mass index (BMI), use of vitamin supplements, etc., see Table 1] but also known variables that affect curve progression (such as bone mineral density, growth stage, and menarchal status). A major problem with the concept that poor vitamin D status [i.e., low concentration of 25(OH)D] is clinically meaningful is that it is unusual to observe signs or symptoms that can be directly attributed to the “low” 25(OH)D concentrations. In fact, patients frequently have 25(OH)D levels in the deficient range without any obvious symptoms or abnormalities in standard serum chemistries. In addition to the well known detrimental effect of vitamin D deficiency on bone, vitamin D deficiency can also have a detrimental effect on skeletal muscle; these include type 2 muscle fiber atrophy and metabolic changes manifest as muscle weakness which has been associated with increased risk of falling (1,2,10,11). Thus, we suggest all studies should attempt to collect bone mineral density, and weight- and age-adjusted measures of muscle strength, which could include grip strength or proximal muscle strength (ability to rise from a sitting position, stair climbing or speed walking) (11). If such measures are normal in the presence of vitamin D insufficiency then that could argue against the clinical relevance of an observation of suboptimal vitamin D status. A measurement of parathyroid hormone (PTH) may be useful in interpreting the 25(OH)D values (12,13,14,15). A feedback relationship between serum ionized calcium, PTH and vitamin D metabolism is well established [as blood calcium levels drop, PTH levels rise and among other effects, stimulate synthesis of 1,25(OH)D] (1,2). Hence, PTH will increase if 25(OH)D is low enough to impact calcium metabolism, although the set point for this may vary from patient to patient (12,13,14,15). Theoretically, if a compensatory increase in PTH is not observed in conjunction with a “low” 25(OH)D, then the clinical significance of the “low” 25(OH)D value may be questionable. While these relationships are well established in adults, a compensatory increase in PTH as 25(OH)D falls below a critical threshold may occur at different thresholds in children or elderly adults (14).

Investigators must carefully select the control group used to compare 25(OH)D concentrations or prevalence of deficiency. This is not easily accomplished but is often directly related to the study hypothesis. For example, if the study hypothesis is that low 25(OH)D is a risk factor for severe pediatric forearm fractures requiring surgical reduction, then a logical control group would be patients with less severe fractures that can be treated conservatively, who are matched for age, sex, BMI, activity level, sun exposure, multivitamin use, etc. It would not be appropriate, for example, to use hospitalized children who may have illnesses that could impact vitamin D status.

## Inconsistent Presentation of Results

Most papers present serum concentrations of 25(OH)D as the group mean ± SD. However, perhaps more important is the distribution of values within the cohort (the percent that are deficient, insufficient and sufficient). Some papers do not report this distribution, so it is not possible to fully interpret the mean 25(OH)D value (see discussion of subgroup analyses below). Box and whisker plots would be an effective way to present these data.

One issue related to the presentation of a distribution of values is the definition of cutoffs defining deficiency. Unfortunately, these vary according to which guidelines are followed, but most are trending toward defining vitamin D deficiency as 25(OH)D <20 ng/mL (Table 2) (16,17,18,19,20,21).

## Failure to Account for Variables Known to Affect the Serum Concentration of 25(OH)D

Interpretation of study results can be influenced by subgroup analysis, and failure to report subgroup results can distort the true findings in a study. It is critically important to carefully characterize the study cohort because many factors can affect the 25(OH)D level in blood (either increasing or decreasing the concentration) (Table 1). Lifestyle factors known to affect 25(OH)D include sun exposure practices such exuberant use of sunscreen, diets low in dairy products and various supplements and medications that influence vitamin D metabolism (multivitamins, calcium supplements with vitamin D, anticonvulsants, etc.). Perhaps the most important patient characteristic that affects 25(OH)D is race/ethnicity. Studies consistently report that nonwhite cohorts (African Americans, Hispanics, Asians) have significantly lower 25(OH)D concentrations than Caucasians. However, the relative contributions of genetics (including skin pigmentation and body fat profiles), socioeconomic status and culture (including diet low in vitamin D, avoidance of sun exposure, etc.) on these findings remains unclear. Another important demographic variable is BMI, as 25(OH)D is lower in obese subjects compared to normal weight subjects. Failure to carefully match controls, or failure to present subgroup analyses can lead to bias in results and misinterpretation. The implications are obvious. For example, if one cohort has a high percentage of patients who take multivitamins that contain a form of vitamin D and the comparative group does not, then the difference in 25(OH)D between them could be affected by the use multivitamins.

Table 3 shows results of a study conducted to document vitamin D status in children with radius fractures (mean age, 9.8±3.4 years; 65% were boys). These previously unpublished data show the potential for misleading reporting of study findings. The total cohort mean is less than the cuttoff of 30 ng/mL that many labs and some guidelines define as vitamin D sufficiency. However, the subgroup of Caucasian subjects had a mean 25(OH)D that was 25% higher (and in the “sufficient” range, >30 ng/mL), compared to all nonwhites [despite no significant difference in BMI, a variable that can affect 25(OH)D concentration]. Thus, we suggest it would be misleading to report only the total group mean 25(OH)D value given this significant subgroup difference. Table 4 shows the covariable confounder of BMI on the risk for severe fracture, in which both a high BMI (classifying patients as obese or overweight) and 25(OH)D deficiency were independent risk factors for having a severe distal radius fractures requiring surgical management. Failure to report and discuss the impact of BMI would be misleading, possibly overweighting the impact of the vitamin D status as risk factor for severe fractures.

## 25(OH)D Assay Issues

Multiple assay systems are available for the measurement of 25(OH)D in blood. These assays can be grouped into two general categories: 1) immune based and 2) chromatography based (ultraviolet or mass spectrometric detection). The mass spectroscopy systems are currently favored as the standard. In fact, the liquid chromatography tandem mass spectroscopy method is used by the Center for Disease Control and Prevention as the reference measurement system. However, these systems require sophisticated equipment and technical expertise and are difficult to automate. Hence, immunoassays are more commonly available.

Unfortunately, there are well described problems with variation and a lack of congruity between different assays systems, with considerable differences in the 25(OH)D concentration reported when the same sample is assayed by different systems (36,37,38,39,40,41,42). Further, there are differences between assays with respect to their ability to measure both 25(OH)D2 and 25(OH)D3 and some assays may cross-react and measure other metabolites. There can be a clinically important difference if one assay system measures 25(OH)D 10-15% lower than another, as it could impact the number of patients reported as deficient. These assay differences may be problematic when attempting to compare studies or do meta-analyses. Thus, it is important to know in detail about the assay system used when evaluating the results of a study (some papers do not report the type of assays used). Efforts are in progress to standardize 25(OH)D assays and to have lab certification of assay systems to mitigate the various issues associated with multiple assays types. However, at the time of writing, such a solution has not been implemented.

## Solutions and Recommendations

Professional societies or journals that publish vitamin D-related studies could help standardize reporting of results by providing guidelines and checklists for ensuring some degree of study standardization. This especially applies to standardizing the presentation of cutoff values for defining deficiency.

## Design

 •    Investigators should state *a priori* what difference between the main study cohort and the comparative group is hypothesized to be clinically relevant to frame later interpretation of obtained results.

•    Investigators should measure and report all variables known to affect 25(OH)D and make adjustments in data, or discuss the absence of such information in a paragraph regarding study limitations and the possible impact on interpretation.

•    Measurements of 25(OH)D should occur over the time interval during which an effect or outcome is expected, not merely at the beginning or end of a study.

•    While it is important for the sake of generalizability to have diversity in study cohorts (and institutional review boards may require it), it may be problematic for vitamin D studies. Study designs should either consider enrolling homogeneous patient groups or anticipate the need for subgroups analyses. The latter would require a randomized block design for prospective studies or matched patient selection in retrospective studies, with large enough sample size to allow for subgroup analyses.

## Presentation of Data

•    The mean 25(OH)D alone does not provide enough information about the true vitamin D status in a cohort. All vitamin D studies should report not only the mean 25(OH)D but also the percent deficiency (the range of values can be eloquently presented using box and whisker plots). However, guidelines defining deficiency are not consistent. We suggest that a solution for minimal standardization is for journals to require that results be presented for a sliding scale of cutoff points, reporting percent deficiency at <10, <20 and <30 ng/mL (as simple bar graphs). Not only would that allow readers to judge for themselves what is true deficiency, but would be beneficial for comparing results between papers and documenting important detail in the literature in the likely event of future adjustments of cutoff values.

## 25(OH)D Assay

•    Details of the assay must be presented in publications, and if possible, whether the lab and assay have been certified per the National Institute of Standards and Technology (an agency of the U.S. Department of Commerce, Gaithersburg, MD), or the Vitamin D Standardization Program of the National Institutes of Health Office of Dietary Supplement.

•    If investigators plan to do long-term data collection or multiple vitamin D studies, they should consider preparing an internal standard that can be remeasured over time or in serial studies to account for assay drift. This can be done by collecting a large sample of blood and then freezing aliquots that can be serially measured. This would be particularly important in multisite studies if a central assay lab could not be used. Reference samples of 25(OH)D can also be purchased commercially. Alternatively, samples can be held until the end of the study and run together as a single batch to avoid issues with assay drift [serum 25(OH)D is generally stable when samples are frozen at -20 degrees centigrade and not affected by multiple freeze-thaw cycles].

## Conclusion

We suggest that guidelines should be available to standardize studies of vitamin D and data presentation to allow for direct comparisons and for high-quality meta-analyses. The authors believe that the suggestions made here are relatively easy to implement. Professional societies and especially journal editorial boards should consider checklists to ensure critical data elements are presented in published vitamin D studies.

## Figures and Tables

**Table 1:**
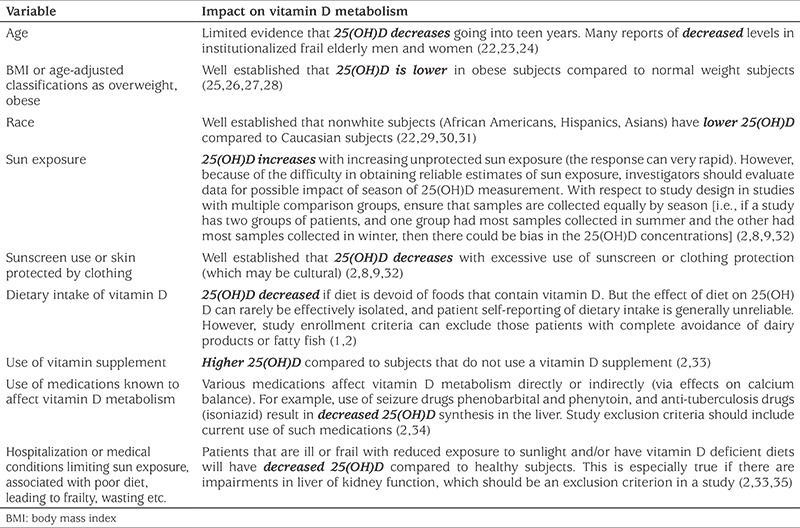
Variables known to affect serum 25(OH)D concentration

**Table 2:**
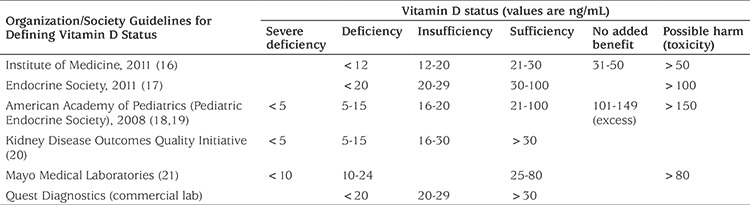
Variation in guidelines defining vitamin D status

**Table 3:**
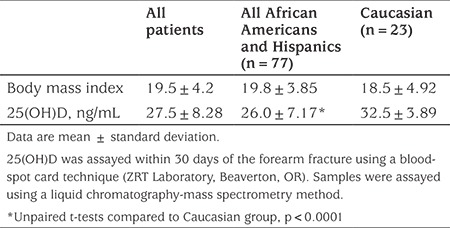
Subgroup effects on reported mean 25(OH)D concentration in 100 children with radius fractures (previously unpublished data)

**Table 4:**
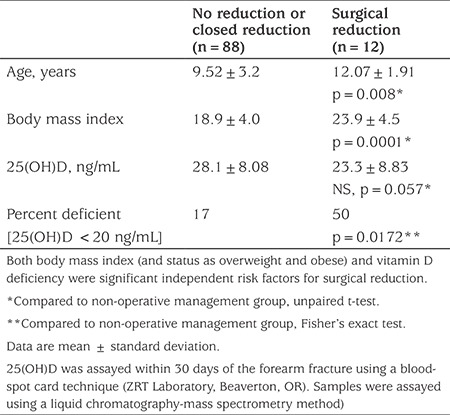
Body mass index as a significant confounder in study results (previously unpublished data)

## References

[1] DeLuca HF (2004). Overview of general physiologic features and functions of vitamin D. Am J Clin Nutr.

[2] Holick MF (2011). Vitamin D: evolutionary, physiological, and health perspectives. Curr Drug Targets.

[3] Balioglu MB, Aydin C, Kargin D, Albayrak A, Atici Y, Tas SK, Kaygusuz MA (2016). Vitamin D measurement in patients with adolescent idiopathic scoliosis. J Pediatr Orthop B.

[4] Batista R, Martins D, Wajchenberg M, Lazaretti M, Puertas E, Terreri M, Hayashi M (2014). Association between vitamin D levels and adolescent idiopathic scoliosis. Columna.

[5] Goździalska A, Jaśkiewicz J, Knapik-Czajka M, Drąg J, Gawlik M, Cieśla M, Kulis A, Zarzycki D, Lipik E (2016). Association of calcium and phosphate balance, vitamin D, PTH, and calcitonin in patients with adolescent idiopathic scoliosis. Spine.

[6] Silva RTE, Fernandes RJR, Ono AHA, Marcon RM, Cristante AF, Barros TEP Filho (2017). Role of different hormones in the pathogenesis and severity of adolescent idiopathic scoliosis. Acta Ortop Bras.

[7] Suh KT, Eun IS, Lee JS (2010). Polymorphism in vitamin D receptor is associated with bone mineral density in patients with adolescent idiopathic scoliosis. Eur Spine J.

[8] Chen TC, Chimeh F, Lu Z, Mathieu J, Person KS, Zhang A, Kohn N, Martinello S, Berkowitz R, Holick MF (2007). Factors that influence the cutaneous synthesis and dietary sources of vitamin D. Arch Biochem Biophys.

[9] Holick MF, Chen TC, Lu Z, Sauter E (2007). Vitamin D and skin physiology: a D-lightful story. J Bone Miner Res.

[10] Hamilton B (2010). Vitamin D and human skeletal muscle. Scand J Med Sci Sports.

[11] Annweiler C, Schott AM, Montero-Odasso M, Berrut G, Fantino B, Herrmann FR, Beauchet O (2010). Cross-sectional association between serum vitamin D concentration and walking speed measured at usual and fast pace among older women: the EPIDOS study. J Bone Miner Res.

[12] Atapattu N, Shaw N, Högler W (2013). Relationship between 25-hydroxyvitamin D and parathyroid hormone in the search for a biochemical definition of vitamin D deficiency in children. Pediatr Res.

[13] Ginde AA, Wolfe P, Cargo CA, Schwartz RS (2012). Defining vitamin D status by secondary hyperparathyroidism in the U.S. population. J Endocrinol Invest.

[14] Hill KM, McCabe GP, Gordon CM, Abrams SA, Weaver CM (2010). An inflection point of serum 25-hydroxyvitamin D for maximal suppression of parathyroid hormone is not evident from multi-site pooled data in children and adolescents. J Nutr.

[15] Valcour A, Blocki F, Hawkins DM, Rao SD (2012). Effects of age and serum 25-OH-vitamin D on serum parathyroid hormone levels. J Clin Endocrinol Metab.

[16] No authors listed (2011.). Dietary reference takes for calcium and vitamin D. Washing DC. The National Academies Press, IOM.

[17] Holick MF, Binkley NC, Bischoff-Ferrari HA, Gordon CM, Hanley DA, Heaney RP, Murad MH, Weaver CM;, Endocrine Society (2011). Evaluation, treatment, and prevention of vitamin D deficiency: an Endocrine Society clinical practice guideline. J Clin Endocrinol Metab.

[18] Wagner CL, Greer FR;, American Academy of Pediatrics Section on Breastfeeding; American Academy of Pediatrics Committee on Nutrition (2008). Prevention of rickets and vitamin D deficiency in infants, children and adolescents. Pediatrics.

[19] Misra M, Pacaud D, Petryk A, Collett-Solberg PF, Kappy M;, Drug and Therapeutics Committee of the Lawson Wilkins Pediatric Endocrine Society (2008). Vitamin D deficiency in children and its management: Review of current knowledge and recommendations. Pediatrics.

[20] No authors listed (2005.). National Kidney Foundation, Inc. Guideline 8. Prevention and treatment of vitamin D insufficiency and vitamin D deficiency in CKD patients. KDOQI Clinical practice guidelines for bone metabolism and disease in children with chronic kidney disease.

[21] Kennel KA, Drake MT, Hurley DL (2010). Vitamin D deficiency in adults: When to test and how to treat. Mayo Clin Proc.

[22] Basatemur E, Horsfall L, Marston L, Rait G, Sutcliffe A (2016). Trends in the diagnosis of vitamin D deficiency. Pediatrics.

[23] Ginde AA, Liu MC, Camargo CA Jr (2009). Demographic differences and trends of vitamin D insufficiency in the US population, 1988-2004. Arch Intern Med.

[24] Mansbach JM, Ginde AA, Camargo CA Jr (2009). Serum 25-hydroxyvitamin D levels among US children aged 1 to 11 years: do children need more vitamin D?. Pediatrics.

[25] Turer CB, Lin H, Flores G (2013). Prevalence of vitamin D deficiency among overweight and obese children. Pediatrics..

[26] Rogers R, Eagle TF, Sheetz A, Woodward A, Leibowitz R, Song M, Sylvester R, Corriveau N, Kline-Rogers E, Jiang Q, Jackson EA, Eagle KA (2015). The Relationship between Childhood Obesity, Low Socioeconomic Status, and Race/Ethnicity: Lessons from Massachusetts. Child Obes.

[27] Carrelli A, Bucivsky M, Horst R, Cremers S, Zhang C, Bessler M, Schrope B, Evanko J, Blanco J, Silverberg SJ, Stein EM (2017). Vitamin D storage in adipose tissue of obese and normal weight women. J Bone Miner Res.

[28] Au LE, Rogers GT, Harris SS, Dwyer JT, Jacques PF, Sacheck JM (2013). Associations of vitamin D intake with 25hydroxyvitamin D in overweight and racially/ethnically diverse US children. J Acad Nutr Diet.

[29] Schleicher RL, Sternberg MR, Lacher DA, Sempos CT, Looker AC, Durazo-Arvizu RA, Yetley EA, Chaudhary-Webb M, Maw KL, Pfeiffer CM, Johnson CL (2016). The vitamin D status of the US population from 1988 to 2010 using standardized serum concentrations of 25-hydroxyvitamin D shows recent modest increases. Am J Clin Nutr.

[30] Parry J, Sullivan E, Cooper A (2011). Vitamin D sufficiency screening in preoperative pediatric orthopedic patients. J Pediatr Orthop.

[31] Minkowitz B, Cerame B, Poletick E, Nguyen JT, Formoso ND, Luxenberg SL, Lee BH, Lane JM;, Morris-Essex Pediatric Bone Health Group (2017). Low vitamin D levels are associated with need for surgical correction of pediatric fractures. J Pediatr Orthop.

[32] Schramm S, Lahner H, Jöckel KH, Erbel R, Führer D, Moebus S;, Heinz Nixdorf Recall Study Group (2017). Impact of season and different vitamin D thresholds on prevalence of vitamin D deficiency in epidemiological cohorts-a note of caution. Endocrine.

[33] Kiebzak GM, Moore NL, Margolis S, Hollis B, Kevorkian CG (2007). Vitamin D status of patients admitted to a hospital rehabilitation unit: relationship to function and progress. Am J Phys Med Rehabil.

[34] Gröber U, Kisters K (2012). Influence of drugs on vitamin D and calcium metabolism. Dermatoendocrinol.

[35] Moore NL, Kiebzak GM (2007). Suboptimal vitamin D status is a highly prevalent but treatable condition in both hospitalized patients and the general population. J Am Acad Nurse Pract.

[36] Atef SH (2018). Vitamin D assays in clinical laboratory: past, present and future challenges. J Steroid Biochem Mol Biol.

[37] Binkley N, Wiebe D (2013). Clinical controversies in vitamin D: 25(OH)D measurement, target concentration, and supplementation. J Clin Densitom.

[38] Carter GD (2010). Accuracy of 25-hydroxyvitamin D assays: Confronting the Issues. Curr Drug Targets.

[39] Le Goff C, Cavalier E, Souberbielle JC, González-Antuña A, Delvin E (2015). Measurement of circulating 25-hydroxyvitamin D: a historical review. Pract Lab Med.

[40] Lai JK, Lucas RM, Banks E, Ponsonby AL;, Ausimmune Investigator Group (2012). Variability in vitamin D assays impairs clinical assessment of vitamin D status. Intern Med J.

[41] Newman MS, Brandon TR, Groves MN, Gregory WL, Kapur S, Zava DT (2009). A liquid chromatography/tandem mass spectrometry method for determination of 25-hydroxy vitamin D2 and 25-hydroxy vitamin D3 in dried blood spots: a potential adjunct to diabetes and cardiometabolic risk screening. J Diabetes Sci Technol.

[42] Sempos CT, Vesper HW, Phinney KW, Thienpont LM, Coates PM;, Vitamin D Standardization Program (VDSP) (2012). Vitamin D status as an international issue: national surveys and the problem of standardization. Scan J Clin Lab Invest Suppl.

